# Results from tandem Phase 1 studies evaluating the safety, reactogenicity and immunogenicity of the vaccine candidate antigen *Plasmodium falciparum* FVO merozoite surface protein-1 (MSP1_42_) administered intramuscularly with adjuvant system AS01

**DOI:** 10.1186/1475-2875-12-29

**Published:** 2013-01-23

**Authors:** Nekoye Otsyula, Evelina Angov, Elke Bergmann-Leitner, Margaret Koech, Farhat Khan, Jason Bennett, Lucas Otieno, James Cummings, Ben Andagalu, Donna Tosh, John Waitumbi, Nancy Richie, Meng Shi, Lori Miller, Walter Otieno, Godfrey Allan Otieno, Lisa Ware, Brent House, Olivier Godeaux, Marie-Claude Dubois, Bernhards Ogutu, W Ripley Ballou, Lorraine Soisson, Carter Diggs, Joe Cohen, Mark Polhemus, D Gray Heppner, Christian F Ockenhouse, Michele D Spring

**Affiliations:** 1 Walter Reed Project, Kenya Medical Research Institute, Kisumu, Kenya; 2 Malaria Vaccine Branch, Walter Reed Army Institute of Research, 2460 Linden Lane, Bldg #503, Silver Spring, USA; 3 GlaxoSmithKline Biologicals, Rue de l’Institut 89, Rixensart, Belgium; 4 US Agency for International Development, 1300 Pennsylvania Avenue, Washington DC, 20523, USA

**Keywords:** Malaria, Vaccine, Merozoite surface protein-1, *Plasmodium*

## Abstract

**Background:**

The development of an asexual blood stage vaccine against *Plasmodium falciparum* malaria based on the major merozoite surface protein-1 (MSP1) antigen is founded on the protective efficacy observed in preclinical studies and induction of invasion and growth inhibitory antibody responses. The 42 kDa C-terminus of MSP1 has been developed as the recombinant protein vaccine antigen, and the 3D7 allotype, formulated with the Adjuvant System AS02A, has been evaluated extensively in human clinical trials. In preclinical rabbit studies, the FVO allele of MSP1_42_ has been shown to have improved immunogenicity over the 3D7 allele, in terms of antibody titres as well as growth inhibitory activity of antibodies against both the heterologous 3D7 and homologous FVO parasites.

**Methods:**

Two Phase 1 clinical studies were conducted to examine the safety, reactogenicity and immunogenicity of the FVO allele of MSP1_42_ in the adjuvant system AS01 administered intramuscularly at 0-, 1-, and 2-months: one in the USA and, after evaluation of safety data results, one in Western Kenya. The US study was an open-label, dose escalation study of 10 and 50 μg doses of MSP1_42_ in 26 adults, while the Kenya study, evaluating 30 volunteers, was a double-blind, randomized study of only the 50 μg dose with a rabies vaccine comparator.

**Results:**

In these studies it was demonstrated that this vaccine formulation has an acceptable safety profile and is immunogenic in malaria-naïve and malaria-experienced populations. High titres of anti-MSP1 antibodies were induced in both study populations, although there was a limited number of volunteers whose serum demonstrated significant inhibition of blood-stage parasites as measured by growth inhibition assay. In the US volunteers, the antibodies generated exhibited better cross-reactivity to heterologous MSP1 alleles than a MSP1-based vaccine (3D7 allele) previously tested at both study sites.

**Conclusions:**

Given that the primary effector mechanism for blood stage vaccine targets is humoral, the antibody responses demonstrated to this vaccine candidate, both quantitative (total antibody titres) and qualitative (functional antibodies inhibiting parasite growth) warrant further consideration of its application in endemic settings.

**Trial registrations:**

Clinical Trials NCT00666380

## Background

A highly efficacious, long-lasting malaria vaccine arguably requires a multistage immunogen that prevents infection or, failing that, impedes development of clinical disease. Towards this end, many of the research efforts within the malaria vaccine development programme at the Walter Reed Army Institute of Research (WRAIR) have focused on developing recombinant protein *Plasmodium falciparum* vaccine antigens which, when mixed with potent adjuvants such as AS01, an Adjuvant System containing 3-*O*-desacyl-4’-monophosphoryl lipid A (MPL) and *Quillaja saponaria* Molina, fraction 21 (QS21) in liposomes, can elicit protective immune responses. Such antigens could then be combined with promising circumsporozoite-based vaccine candidates such as adjuvanted RTS,S which is currently in Phase 3 trials [1,2].

Merozoite surface protein-1 (MSP1), found on the surface of merozoites, is one such protein, with the 42 kDa C-terminal fragment developed as the vaccine antigen. Since the 42 kDa fragment contains known B- and T-cell cell epitopes [3,4], a MSP1_42_ vaccine antigen may be capable of conferring protection mediated by providing antigen-specific T-cell help for B-cells and antibody production as well as by stimulating effector T-cells and the secretion of lymphokines. Both MSP1_42_ and MSP1_19_ are established targets of protective immunity in animal models, and in both murine and non-human primate studies the protection afforded by vaccination with MSP1 is strain specific [5,6]. The 3D7 allele of MSP1_42_ has been under clinical development at WRAIR for over 10 years [7] with accumulated safety and reactogenicity data from two trials conducted at WRAIR [8], unpublished data J Cummings] and four trials in endemic areas in Mali and Kenya [9-12]. In all studies, the vaccine candidate MSP1_42_ formulated in AS02 (an Adjuvant System containing MPL and QS21 in an oil-in-water emulsion) was shown to have an acceptable safety profile and be immunogenic. The Phase 1 dose-escalation study conducted in 15 malaria-naïve adults at WRAIR in 2001 demonstrated induction of low levels of functional antibodies able to inhibit growth of homologous 3D7 parasites in a growth inhibition assay (GIA) [8]. The subsequent Phase 2 paediatric malaria vaccine study conducted in Kenya failed to demonstrate protective efficacy against clinical disease [12], but significant protection was detected against a subset of parasites with allelic homology with the vaccine [unpublished observations, C Ockenhouse].

Evidence from both preclinical and clinical studies suggests that without significant cross-reactivity, vaccination with a single MSP1 allotype may not achieve broad efficacy and actually may contribute to selection of alternate alleles [13]. Therefore, researchers at WRAIR also developed an MSP1_42_ protein antigen based on the FVO sequence. Preclinical studies have shown improved immunogenicity of the FVO antigen as compared to the 3D7 antigen in terms of antibody titres as well as growth inhibitory activity of antibodies against both heterologous and homologous parasites [unpublished data, E Angov]. Active vaccination of *Aotus nancymai* monkeys with *Escherichia coli*[14,15] or baculovirus-expressed [16-18] adjuvanted, recombinant FVO MSP1_42_ induces a significant homologous protection. Molecular typing of *P. falciparum* parasites from East and West Africa indicate that >90% of the dominant circulating alleles are QKNG and EKNG, i e, FVO-like and CAMP-like [unpublished data, C Ockenhouse]; therefore, a vaccine that is based on the FVO allotypes of the MSP1_42_ vaccine antigen may stand a better chance of inducing protection in populations living in endemic areas. Conducting these two vaccine trials back-to-back in the USA and Kenya allowed for assessment and comparison of the safety of the FVO candidate in two different populations, as well as the capability of the formulation to induce potentially protective antibody responses.

## Methods

### Study design and population

Two first-in-human clinical studies were conducted to test WRAIR’s FVO MSP1_42_ recombinant protein antigen (a vaccine antigen designated FMP010, for “falciparum malaria protein #10”, herein referred to as MSP1_42_) in 0.5 millilitres (mL) of the AS01 adjuvant System: one at the Clinical Trials Center (CTC) at WRAIR in Silver Spring, Maryland, USA and the other six months later at US Army Medical Research Unit in Kenya (USAMRU-K) at the KEMRI/Walter Reed Project’s Muriithi Wellde Clinical Research Centre, Kombewa, Kenya. The US Phase 1 open-label dose- escalation study enrolled 26 malaria-naïve adults: six subjects to receive approximately 10 μg dose MSP1_42_ antigen in 0.5 mL AS01, and 20 subjects to receive 50 μg MSP1_42_ antigen in 0.5 mL AS01. A Safety Monitoring Committee (SMC) reviewed all safety data after the first vaccination in the 10 μg dose group prior to progression to the 50 μg group as well as after the second vaccination with 50 μg MSP1/AS01 before moving forward to the Kenya study. The Kenya study was a Phase 1, double blind, randomized controlled study in which 30 malaria-exposed adults were randomized in a 2:1 fashion, 20 of whom received 50 μg of the MSP1_42_ antigen adjuvanted in 0.5 mL of AS01 while 10 received *Rabipur*® rabies vaccine (Novartis, Basel, Switzerland). In both studies, the vaccination schedule was at 0, 28 and 56 days with intramuscular route of administration in the deltoid muscle of the non-dominant arm.

The primary objective was to assess the safety and reactogenicity of the MSP1_42_/AS01 candidate malaria vaccine in healthy adults. This was measured by the following endpoints: occurrence and intensity of solicited symptoms on day of vaccination and days 1 through 7 after each vaccination, occurrence and intensity of unsolicited symptoms over a 30-day follow-up period (day of vaccination plus 29 subsequent days) after each vaccination and occurrence of serious adverse events (SAEs) during the study period. The secondary objective was to measure and compare, by quantifying parasite lactate dehydrogenase (pLDH) in a growth inhibition assay (GIA), the functional humoral immune responses induced by MSP1_42_/AS01. In the US study, these immune responses were additionally compared to the humoral immune response previously generated to MSP1_42_ 3D7 allele administered with the AS02 Adjuvant System with the endpoint being percent parasite growth inhibition in GIA against homologous (FVO) and heterologous (3D7) parasites at baseline and post-third vaccination. The tertiary objective was to measure, by enzyme-linked immunoabsorbent assay (ELISA) as performed by the reference centre Malaria Serology Laboratory (MSL) at WRAIR, the humoral immune response induced by MSP1_42_/AS01. Additional tertiary endpoints were titres of antibodies to MSP1_42_ of FVO, 3D7, and CAMP (FUP) antigens as determined by ELISA.

Inclusion criteria included healthy, non-pregnant adults able to participate for the length of the study. Exclusion criteria included prior receipt of any investigational malaria vaccine, vaccine containing either QS-21 or MPL or both, use of any investigational or non-registered drug or vaccine other than the study vaccine within 30 days preceding the first dose of study vaccine, administration of chronic immunosuppressants or other immune-modifying drugs within six months of vaccination, any confirmed or suspected immunosuppressive or immunodeficient condition or family history of congenital or hereditary immunodeficiency, chronic or active neurologic disease including seizure disorder, history of splenectomy, acute or chronic, clinically significant pulmonary, cardiovascular, hepatic or renal functional abnormality, as determined by physical examination or abnormal baseline laboratory screening tests, acute disease at the time of enrolment, hepatomegaly, right upper quadrant abdominal pain or tenderness, administration of immunoglobulins and/or any blood products within the three months preceding the first dose of study vaccine or planned administration during the study period, pregnant or lactating female, suspected or known current alcohol abuse/drug abuse, any history of allergic reaction or anaphylaxis to previous vaccination, inability to make follow-up visits or complete diary cards, allergy to kanamycin, nickel, or imidazole. For the US study, any past history of malaria or planned travel to malarious areas during the study period was exclusionary. All subjects gave written informed consent under non-coercive means. Both studies were conducted under Good Clinical Practices, were approved by the WRAIR Institutional Review Board and the United States Army Medical Research and Materiel Command Human Subjects Research Review Board, and filed under the US Food and Drug Administration Investigational New Drug (IND) application number 13638. In addition, the Kenya study was reviewed by the Kenya Medical Research Institute Ethical Review Committee.

### Vaccine

The MSP1_42_ antigen is the C-terminal 42-kDa portion of the merozoite surface protein-1 (MSP1) from the FVO strain of *P. falciparum* expressed in and purified from BL21 DE3 cell *E. coli* then lyophilized and bottled under cGMP conditions at the WRAIR BioProduction Facility BPF) [7]. MSP1_42_ is a single polypeptide encoding a codon-harmonized sequence of 371 amino acids consisting of 16 non-MSP1 amino acids fused to the N-terminus of a 355 amino acid C- terminal MSP1 (representing base pair (bp) 3834–4898 from the wild type MSP1 FVO sequence, Genbank Accession number: X03371, encoding amino acids 1333–1688). The 16 non-MSP1 amino acids comprise an N-terminal extension from the native MSP1_42_ encoding the translation initiation codon, plus six histidine residues for Ni^+2^-NTA affinity chromatography and a series of repeating glycine, serine residues for flexible extension of the polyhistidines from the body of the protein. Approximately 60 μg of MSP1_42_ (FVO) antigen, production lot number 1157, was packaged in sterile, single dose vials, which then underwent lyophilization. Vials were maintained at the BPF, stored at 2-8°C in a monitored refrigerator.

The antigen was reconstituted with the Adjuvant System AS01 (GlaxoSmithKline Biologicals, Rixensart, Belgium), the main components of which are 50 μg of MPL, 50 μg QS21 (Antigenics, New York, NY, USA) and liposomes. One vial of AS01 provides a dose volume of 0.5 mL of Adjuvant System. Both antigen and Adjuvant System were kept at 2-8°C in monitored refrigerators, and on vaccination days, the vials were placed on wet ice no longer than four hours and mixed at the time of intramuscular administration. For the 10 μg dose, five vials of AS01 were mixed with one vial of MSP1_42_ antigen and 0.5 mL withdrawn for injection, while for the 50 μg dose, one vial of AS01 was formulated with one vial of antigen.

### Safety and reactogenicity

Solicited symptoms included local adverse events (pain, erythema and swelling) and systemic adverse events (fever, nausea, headache, malaise, myalgia, fatigue, and arthralgia). All symptoms were graded on a scale to indicate degree of functional impairment (Grade 0: no impairment, Grade 1: easily tolerated, Grade 2: interferes with daily activity, Grade 3: prevents daily activity) except for injection site erythema and swelling, which were graded as a physical measurement taken at the greatest diameter of involvement (Grade 0: 0 mm, Grade 1: >1 - < 20 mm, Grade 2: >20 - < 50 mm, Grade 3: > 50 mm), and fever, which was graded on the following scale of oral temperature: Grade 0: <37.5°C, Grade 1: >37.5- < 38°C, Grade 2: >38- < 39°C, Grade 3: >39°C. In the US study, subjects were seen in clinic on day 1, 2, 3 and 7 for this assessment; a diary card was also kept on days 0–7. Kenyan subjects were seen by a study clinician on day 0, 3, and 7; no diary cards were kept. Haematologic and biochemical tests for safety were collected on days 0, 14, 28, 42, 56 and 70, and again at three months after last vaccination. Serious adverse events were collected throughout the entire study period. In order to optimize medical care and adverse event data collection in the Kenya study, volunteers were provided with an identification card with emergency medical contacts and had access to the clinical trial centre for medical care 24 hours a day for the duration of the study with provisions for appropriate specialist referral.

### Immunogenicity

#### Antibody titre by ELISA

IgG responses to the *P. falciparum* MSP1_42_ FVO antigen were measured using standard ELISA methodologies at the Malaria Serology Lab (MSL) at WRAIR. Briefly, plates were coated with 100 μ L/well of MSP1_42_ antigen at a concentration of 1.0 μg/mL, placed inside a humidity chamber and incubated overnight (16–20 hrs) at 4^o^C. Plates were washed four times with 1X PBS (pH 7.4) containing 1% Tween-20 and blocked with 0.5% boiled casein (Sigma, St. Louis, MO, USA). Plates were washed four times with 1X PBS solution between all subsequent steps except the development reaction. Serum samples from vaccinees were serially diluted on each plate and incubated at 22°C for 2 hrs. Peroxidase-labelled goat anti–human IgG (KPL, Gaithersburg, MD, USA) was added to each well at a 1:4,000 dilution and incubated for 1 hr at 22°C. ABTS Peroxidase substrate (KPL, Gaithersburg, MD, USA) was added to induce reaction development. At the end of 1-hr incubation at 22°C, a stop solution (20% sodium dodecyl sulphate) was added and the plates were read using a Spectromax340PC plate reader. The absorbance at 414 nm was determined for each well and these data were applied to a fourparameter logistic curve using SoftMax 4.8 (Molecular Devices, Sunnyvale, CA, USA). The serum titre was defined as the serum dilution at which the optical density (OD) was equal to 1.0. The geometric mean antibody titre at each time point was determined with 95% confidence intervals.

#### Growth inhibitory assay (GIA)

Serum samples were dialysed in 50K MW cutoff mini-dialysis units (MWCO Slide-A-Lyzer Mini-Dialysis Units from Pierce, Rockford, IL) to remove any potential anti-malarial drugs [19] and then heat inactivated for 20 min at 56°C. Cooled serum samples were pre-absorbed with 5 μL of human red blood cells (RBC), at 50% haematocrit per 100 μL of serum for 1 hr and tested at 20% serum concentration for growth inhibition by measuring pLDH activity [20]. Parasitized RBC (pRBC) cultures of both the FVO allele (homologous) and the 3D7 allele (heterologous) at the early schizont stage were set up with pre-immune and immune sera at a 0.3% parasitaemia and 1% haematocrit. Assay plates (384 well plates) (Spectraplates, Perkin Elmer, Boston, MA, USA) were sealed in bags containing 2.5% CO2, 2.5% O2, 90% N2 and incubated for 40 or 48 hrs (cycle time of 3D7 and FVO parasites, respectively). Cultures were then harvested by adding 80 μL per well of PBS and spinning plates for 10 min at 10,000 g. Once completed, 80 μL of supernatant were removed and cell pellets frozen at −30°C until analysis. To measure the amount of pLDH activity, a substrate buffer containing 0.1 M Tris HCl, 50 mM Sodium-L-lactate, 0.255 Triton-X, 10 mg NBT, 10 μg/mL 3-Acetylpyridine, and 10 U/mL diaphorase from *Clostridium klyiveri* (all reagents from Sigma, St. Louis, MO, USA) was added to the plates. Colorimetric measurement at 650 nm was done after 30 min of reaction time using the SpectraMax Plus 384 spectrophotometer (Molecular Devices, Sunnyvale, CA, USA). Pre-vaccination samples were run in parallel with the post-vaccination samples, and the calculation of growth inhibition was determined by using the formula: % inhibition = [1-[(OD immune serum –OD RBC)/(OD pre-immune serum – OD RBC)]] × 100. Subjects demonstrating ≥15% activity were considered responders. This value is based on the cutoff values established previously from MSP1 vaccine studies [8] and is a conservative estimate of non-specific, assay related background inhibition.

#### Antibody fine specificity by ELISA

Fine specificity of antibody responses was assessed using recombinant MSP1_42_ FVO, MSP1_42_ 3D7 and MSP1_42_ CAMP. Microtiter plate (Immulon-2 HB; Thermo Labsystems, Franklin, MA, USA) wells were coated with 0.8 pmol of recombinant antigen in 100 μL of PBS and incubated overnight at 4°C followed by blocking for 1 hr at room temperature with 300 μL of blocking buffer (1% Fraction V BSA in PBS). Sera were diluted serially with blocking buffer and incubated for 2 hrs at room temperature. Plates were washed four times with wash buffer (0.05% Tween 20, 0.0025% Chlorohexidine in PBS) using a microplate washer (Skan-Washer 300 version B, Skatron Instruments, Sterling, VA, USA). Goat anti-human IgG (H + L)-alkaline phosphatase conjugate diluted to 1:1,000 (Promega, Madison, WI, USA) was added to each well and incubated at room temperature for 1 hr. *p*-Nitrophenyl phosphate substrate (Sigma, St. Louis, MO, USA), was added to each well and the OD405 nm was measured after 60 min with a microplate reader (Molecular Devices, Spectra-Max Plus 384, SoftMax Pro software). The midpoint antibody titre was calculated as the serum dilution that produced 1.0 optical density 247 (OD) unit in the ELISA assay. For analyses comparing MSP1_42_ fragment-specific antibody titres, the coating antigens were normalized to one another.

#### Statistics

Safety data is descriptively reported and was compiled by RTI International (Research Triangle Park, NC, USA). SAS v9 was used for statistical analysis of humoral immunogenicity data with the exception of day 0 versus day 70 GIA comparison in Kenyan adults (Minitab). A mixed model with unstructured covariance structure is used to compare geometric means of ELISA titres between two dosage groups. Kenward-Rogers’s method is used to estimate the degree of freedom (DF). Post-hoc testing includes examination of the group effect at each time point, and the p value is adjusted using the Tukey’s method for multiple comparisons. Baseline value is compared prior to the model fitting to ensure the comparability between the experimental groups, and excluded from the model. Comparisons of inhibitory activity by serum GIA and allelic ELISA analysis were conducted using a two sample *t*-test. All data were log transformed to stabilize the variance. The distributions of the transformed data were checked to be approximately normal before analysis.

## Results

### Participant flow and demographics

**Figure 1:**
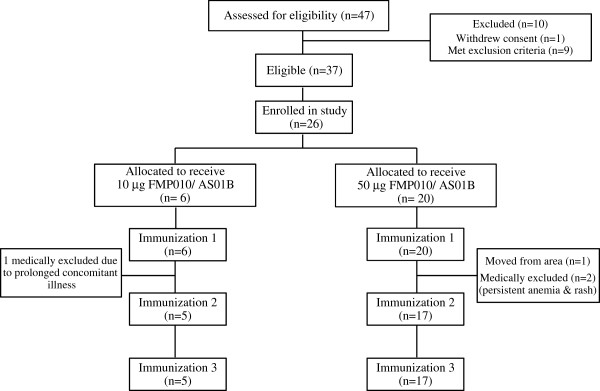
Subject flow chart for Phase 1 study in the USA.

**Figure 2:**
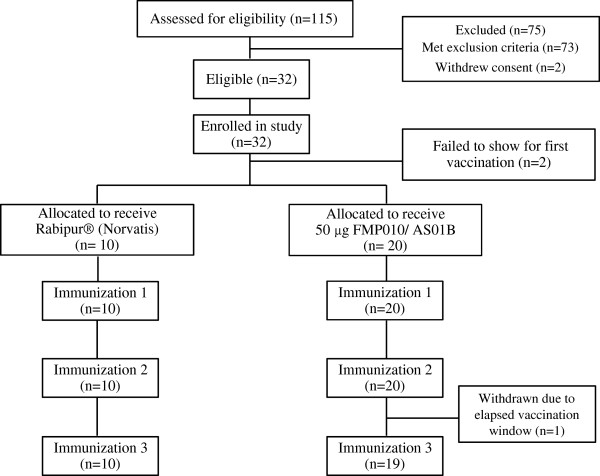
Subject flow chart for Phase 1 study in Kenya.

In Figure 1, the study flow diagram and the number of screened, enrolled and withdrawn subjects in the US study are presented. Approximately 70% of the enrolled subjects were female with the mean age of 32 years. Thirty-nine percent of the subjects reported Caucasian as race/ethnicity while 31% identified as African-American, 12% Hispanic, 8% Native American/Alaskan, 8% other and 4% Asian. Recruitment and vaccination of six subjects in the 10 μg group began in April 2008 with vaccinations for the 50 μg group starting three weeks later after safety review. One subject in the low-dose group was withdrawn after the first vaccination due to a prolonged intercurrent illness, and in the high-dose group, three subjects were lost after the first vaccination. One left the Washington DC area, and the remaining two were withdrawn by the principal investigator (PI), both for non-serious adverse events that are more fully described in the safety section. For the Kenya study, a similar flow diagram is presented in Figure 2. Forty-six percent of the enrolled subjects were female with a mean age of 33 years. All the participants were African with 90% being of Luo ethnicity and 10% of Luhya ethnicity. Recruitment began February 2009 with all vaccinations administered by April 2009. One subject withdrew consent after the third vaccination for personal reasons unrelated to adverse events.

### Safety and reactogenicity

Tables 1, 2 and 3 summarize the local and systemic solicited adverse events (AEs) detected after vaccination in both studies. Regarding local AEs, in the US study, pain, redness and swelling occurred at similar rates for both 10 and 50 μg doses, whereas in the Kenya study, pain was the most common local adverse event following vaccination with both MSP1_42_/AS01 and the rabies vaccine. Most local events for both studies were mild to moderate in severity and generally resolved within 48–72 hours of vaccination, although in the US study, swelling of grade 3 severity was not uncommon in both dose groups. In Kenya, only one female subject experienced grade 3 swelling and redness following the receipt of the third dose of the MSP1_42_/AS01 vaccine. The systemic AEs occurring the most frequently in both studies were fatigue, malaise, headache and myalgia. Overall, the systemic events began approximately 12 hours after vaccination, lasting 24–36 hours. In the US study for both 10 and 50 μg doses, there was a slight increase in severity and frequency of both local and systemic events from the first to second vaccination but no further increase with the third. In the Kenya study there was no consistent increase or decrease in systemic AEs with either the MSP1_42_/AS01 or rabies vaccine. No vaccine-related serious adverse events (SAEs) occurred during either study.

**Table 1:** Solicited adverse events post-vaccination (day 0–7) in the Phase 1 US study

	10 μg MSP142/AS01 (N = 6*)			50 μg MSP142/AS01 (N = 20*)		
	**G1**	**G2**	**G3**	**G1**	**G2**	**G3**
**Pain**						
**Immunization 1**	3	2	0	10	6	2
**Immunization 2**	1	2	1	7	9	1
**Immunization 3**	3	1	0	10	2	3
**Redness**						
**Immunization 1**	1	0	1	3	1	4
**Immunization 2**	1	0	1	1	2	4
**Immunization 3**	2	0	1	3	2	3
**Swelling**						
**Immunization 1**	0	1	1	0	2	8
**Immunization 2**	0	1	2	3	2	6
**Immunization 3**	1	0	3	1	3	10
**Fever**						
**Immunization 1**	2	0	0	1	1	1
**Immunization 2**	1	2	0	4	4	2
**Immunization 3**	2	0	0	3	5	0
**Fatigue**						
**Immunization 1**	1	0	0	6	4	1
**Immunization 2**	1	1	1	5	7	2
**Immunization 3**	1	2	0	7	3	3
**Nausea**						
**Immunization 1**	0	0	0	1	0	0
**Immunization 2**	1	1	0	1	1	0
**Immunization 3**	0	0	0	0	1	1
**Headache**						
**Immunization 1**	2	1	0	3	3	1
**Immunization 2**	3	0	0	3	6	2
**Immunization 3**	1	2	0	2	6	2
**Malaise**						
**Immunization 1**	1	0	0	4	1	2
**Immunization 2**	0	1	2	3	6	4
**Immunization 3**	0	2	0	4	5	3
**Myalgia**						
**Immunization 1**	0	0	0	1	2	1
**Immunization 2**	2	0	0	4	5	2
**Immunization 3**	0	2	0	6	2	3
**Joint Pain**						
**Immunization 1**	0	0	0	2	2	0
**Immunization 2**	0	1	0	3	2	2
**Immunization 3**	0	2	0	4	4	1

**Table 2:** Solicited adverse events post-vaccination (day 0–7) in the Phase 1 Kenya study

	50 μg MSP142/AS01 (N = 20*)			Rabies comparator (N = 10*)		
	**G1**	**G2**	**G3**	**G1**	**G2**	**G3**
**Pain**						
**Immunization 1**	14	6	0	6	0	0
**Immunization 2**	12	7	0	5	0	0
**Immunization 3**	10	4	0	4	0	0
**Redness**						
**Immunization 1**	1	0	0	0	0	0
**Immunization 2**	3	0	0	2	0	0
**Immunization 3**	0	0	1	0	0	0
**Swelling**						
**Immunization 1**	6	0	0	2	0	0
**Immunization 2**	3	0	0	2	0	0
**Immunization 3**	1	0	1	1	0	0
**Fever**						
**Immunization 1**	0	0	0	0	0	0
**Immunization 2**	1	0	0	0	0	0
**Immunization 3**	1	0	0	0	0	0
**Fatigue**						
**Immunization 1**	2	3	0	1	0	0
**Immunization 2**	1	1	0	1	0	0
**Immunization 3**	2	0	0	0	0	0
**Nausea**						
**Immunization 1**	3	0	0	1	0	0
**Immunization 2**	0	1	0	0	0	0
**Immunization 3**	0	0	0	0	0	0
**Headache**						
**Immunization 1**	8	4	0	1	0	0
**Immunization 2**	3	0	1	0	0	0
**Immunization 3**	4	2	0	0	1	0
**Malaise**						
**Immunization 1**	2	1	0	0	0	0
**Immunization 2**	2	3	0	0	0	0
**Immunization 3**	1	1	0	0	0	0
**Myalgia**						
**Immunization 1**	1	0	0	1	0	0
**Immunization 2**	1	2	0	0	0	0
**Immunization 3**	1	0	0	0	0	0
**Joint Pain**						
**Immunization 1**	2	1	0	1	0	0
**Immunization 2**	1	2	0	0	0	0
**Immunization 3**	1	0	0	1	0	0

**Table 3:** **Summary of solicited adverse events post-vaccination (day 0–7) for subjects vaccinated with 50 μg MSP1**_**42**_**/AS01**

				
**Phase 1 US: 50 μg MSP142/AS01 (N = 54**^**#**^**) n(%)**				
	G1	G2	G3	Any
**Pain**	27 (*50%*)	17 *(31%*)	6 (*11%*)	50 (*93%*)
**Redness**	7 (*13%*)	5 (*9%*)	11 (*20%*)	23 (*43%*)
**Swelling**	4 (*7%*)	7 (*13%*)	24 (*44%*)	35 (*65%*)
**Fever**	7 (*13%*)	10 (*19%*)	3 (*6%*)	20 (*37%*)
**Fatigue**	18 (*33%*)	14 (*26%*)	6 (*11%*)	38 (*70%*)
**Nausea**	2 (*4%*)	2 (*4%*)	1 (*2%*)	5 (*9%*)
**Headache**	8 (*15%*)	15 *(28%*)	5 (*9%*)	28 (*52%*)
**Malaise**	11 (*20%*)	12 (*22%*)	7 (*13%*)	30 (*56%*)
**Myalgias**	11 (*20%*)	9 (*17%*)	6 (*11%*)	26 (*48%*)
**Joint Pain**	9 (*17%*)	8 (*15%*)	3 (*6%*)	20 (*37%*)
**Phase 1 Kenya: 50 μg MSP142/AS01 (N = 59**^**#**^**) n(%)**				
	G1	G2	G3	Any
**Pain**	36 (*61%*)	17 (*29%*)	0 (*0%*)	53 (*90%*)
**Redness**	4 (*7%*)	0 (*0%*)	1 (*2%*)	5 (*8%*)
**Swelling**	10 (*17%*)	0 (*0%*)	1 (*2%*)	11 (*19%*)
**Fever**	2 (*3%*)	0 (*0%*)	0 (*0%*)	2 (*3%*)
**Fatigue**	5 (*8%*)	4 (*7%*)	0 (*0%*)	9 (*15%*)
**Nausea**	3 (*5%*)	1 (*2%*)	0 (*0%*)	4 (*7%*)
**Headache**	15 (*25%*)	6 (*10%*)	1 (*2%*)	22 (*37%*)
**Malaise**	5 (*8%*)	5 (*8%*)	0 (*0%*)	10 (*17%*)
**Myalgias**	3 (*5%*)	2 (*3%*)	0 (*0%*)	5 (*8%*)
**Joint Pain**	4 (*7%*)	3 (*5%*)	0 (*0%*)	7 (*12%*)

*N represents the number of volunteers enrolled and who received the first vaccination. For the US Phase 1, five volunteers received the second and third vaccination with 10 μg MSP142/AS01. For the 50 μg MSP142/AS01 group, 20 received the first vaccination and 17 the second and third vaccination (^#^total of 54). In the Kenya Phase 1 study, 20 volunteers received the first and second vaccination with 50 μg MSP142/AS01 and 19 the third vaccination (^#^total of 59) while 10 volunteers received all three rabies vaccinations. Each volunteer is only counted one time per vaccination time point and the highest recorded grade for that AE is reported. G1 = Grade 1, G2 = Grade 2, G3 = Grade 3. Grading system describes degree of functional impairment (Grade 0: no impairment, Grade 1: easily tolerated, Grade 2: interferes with daily activity, Grade 3: prevents daily activity). Injection site redness and swelling grading were as follows: Grade 0: 0 mm, Grade 1: >1 - < 20 mm, Grade 2: >20 - < 50 mm, Grade 3: > 50 mm. Fever was graded by oral temperature: Grade 0: <37.5°C, Grade 1: >37.5 - < 38°C, Grade 2: >38 - < 39°C, Grade 3: >39°C.

In the US study, one female subject vaccinated with 50 μg MSP1_42_/AS01, developed several pruritic, erythematous maculopapules on the right (vaccinated) arm one day post-immunization. The lesions were physically consistent with insect bites, and the subject reported staying in a hotel the night before that may have had infestations. These lesions spread to other extremities and persisted at varying intensities. Biopsy of a lesion by a dermatologist offered a differential diagnosis of arthropod assault *versus* drug reaction. All safety laboratory tests were normal except for increased eosinophil counts, which were 628 cells/μL immediately prior to the first vaccination, which peaked two weeks post-vaccination at 1,020 cells/μL. The subject was withdrawn from further vaccinations, and the rash and eosinophilia completely resolved without sequelae by five weeks post-vaccination. A second subject (male) was withdrawn due to persistent anaemia that began on the day of, but prior to, first vaccination and persisted over the next two weeks, ranging from 11.9-12.6 g/dL (grade 1). The subject was seen by a gastroenterologist and an upper and lower gastrointestinal endoscopy with biopsies was performed; all findings were normal. A haematologist reviewed the chart, and a possible diagnosis of alcohol-induced bone marrow suppression was made, and the subject was withdrawn from the study.

### Humoral immunogenicity

#### Anti-MSP-1_42_ antibodies titres by ELISA

**Figure 3:**
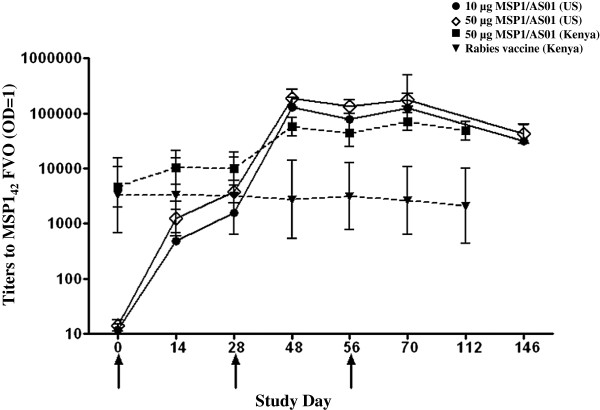
**Antibody titres to MSP1**_**42 **_**FVO by ELISA.** Log ELISA OD 1.0 titres are reported as the geomean and 95% confidence intervals (CIs). Hatched line and filled-in squares are Kenyan subjects receiving 50 μg MSP1_42_ FVO/AS01, hatched lines and triangles are Kenyan subjects receiving rabies vaccine, solid lines and filled in circles are US subjects receiving 10 μg MSP1_42_ FVO/AS01 and solid line with open diamond are US subjects receiving 50 μg MSP1_42_ FVO/AS01. X axis is day of study and Y axis is dilution reporting an OD =1.0 of MSP1_42_ specific antibody. Plate antigen was the WRAIR *E. coli* expressed recombinant protein, MSP1_42_ FVO.

The vaccine specific MSP1_42_ FVO ELISA assay was performed by the MSL and used MSP1_42_ FVO as the plate antigen. The geometric mean titres to both the 10 and 50 μg doses in the US study and the 50 μg dose and rabies control in the Kenya study were calculated and plotted on log scale with 95% confidence intervals (CIs) as shown in Figure 3. No statistical model is fitted between Kenya and US studies since the baseline antibody values are significantly different.

For the US study, a longitudinally borderline significant difference in geometric mean of ELISA titres between the two dosage groups (p = 0.0510) was observed. As expected there was no difference in geometric mean ELISA titres between the two dosage groups on day 0, the baseline (p = 0.9560). There were only minor statistically significant differences in point-wise titres whereby the titres for the 50 μg dose group were slightly higher than 10 μg group: on day 28 (adjusted p = 0.0676) and day 56 (adjusted p = 0.0477). In Kenyan adults, by longitudinal analysis, the antibody titres induced by the 50 μg dose of MSP1_42_/AS01 were greater than for the rabies group, (p < 0.0001). While for point-wise comparisons, the MSP1_42_/AS01 titres were higher than rabies at all time points tested (p < 0.0001), except for day 0 (p = 0.64), day 14 (p = 0.09) and day 28 (p = 0.11).

#### Functional antibody responses (GIA)

**Figure 4:**
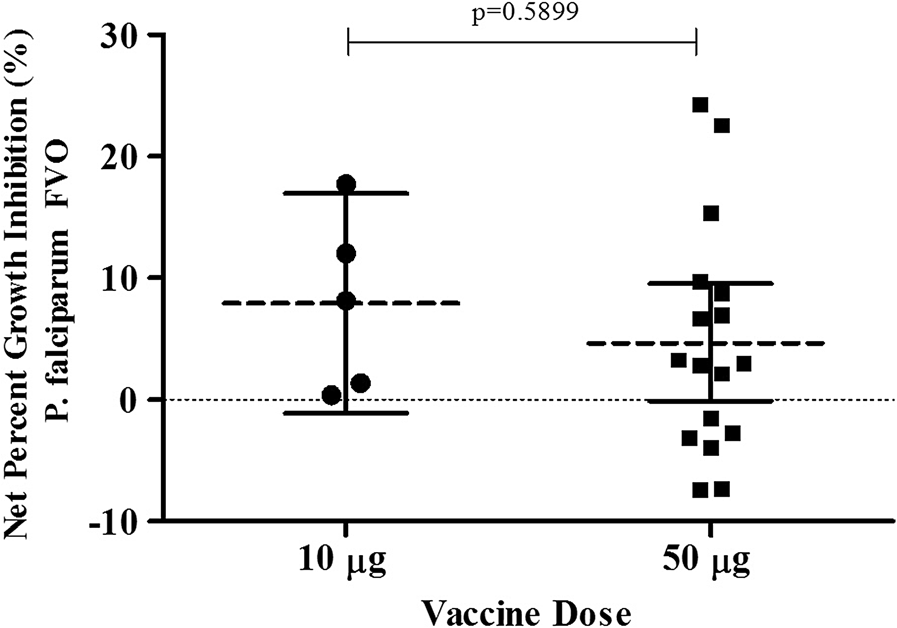
**Results of serum GIA for 10 μg and 50 μg MSP1**_**42 **_**(FVO)/AS01 vaccine in malaria-naïve adults.** Percent parasite growth inhibition against *P. falciparum* FVO clone parasites from the US MSP1_42_ FVO/AS01 study. The net percent growth inhibition of pre-and post third immunization sera obtained for 10 μg and 50 μg MSP1_42_/AS01 dose subjects is shown tested at 20% (v/v, final). Dashed horizontal lines indicate the median response, while the filled bars indicate the 25 and 75% quartiles. Individual responses are represented as either filled circles for the 10 μg or filled squares for the 50 μg dose subjects. P value calculated from two-tailed *t*-test.

Measurement of functional anti-MSP1_42_ antibodies from immunized subjects was carried out with both the homologous FVO, and heterologous 3D7, *P. falciparum* strains (Figure 4). The net percentage inhibition is reported since inhibition detected on day 0 samples are subtracted from the responses measured for day 70 (see equation in Methods). When sera from vaccinated malaria-naïve subjects were tested by GIA against homologous parasites, there was no significant difference in median percent inhibition for the 10 μg dose group (9%) and the 50 μg dose group (5%), (p = 0.5899) with only three subjects having inhibitory activities above a previously determined cut off of 15%. There was no detectable inhibition demonstrated against heterologous 3D7 parasites using this same assay (data not shown).

**Figure 5:**
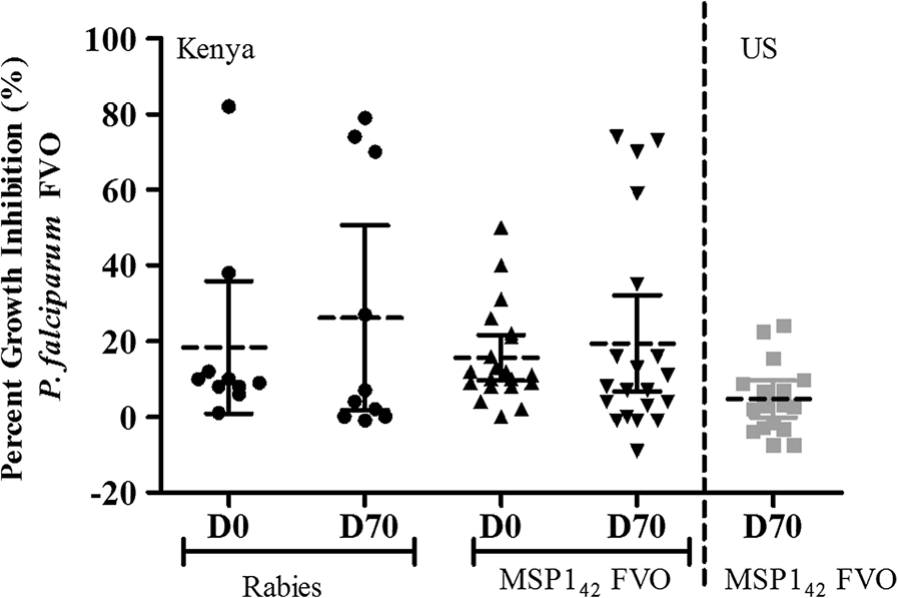
**Results of serum GIA against *Plasmodium falciparum *FVO parasites for subjects receiving rabies vaccine and the 50 μg MSP1**_**42 **_**(FVO)/AS01 vaccines in both Phase 1 studies.** Percent parasite growth inhibition against the *P. falciparum* FVO clone parasites, comparing serum antibody responses obtained from Kenyan adults and US naïve subjects immunized with the 50 μg dose of MSP1_42_ FVO/AS01. Left panel are the day 0 and post third immunization, day 70, responses from Kenya adults either receiving the rabies comparator control vaccine or the 50 μg dose of MSP1_42_ FVO/AS01. Right panel are the net percent growth inhibitory responses for the US subjects receiving the 50 μg dose of MSP1_42_ FVO/AS01. Dashed horizontal lines indicate median responses, while the filled lines indicate the 25 and 75% quartiles. Individual responses are represented as either filled circles for the Kenyan adults receiving the rabies vaccine, filled triangles for the Kenyan adults receiving the MSP1_42_ FVO/AS01 vaccine or filled gray squares for the US naïve subjects receiving the 50 μg dose MSP1_42_ FVO/AS01.

As shown in Figure 5, in Kenyan adults there was no statistically significant difference in GIA activity between the MSP1_42_/AS01 group and the rabies group (p = 0.3108). On day 70, only five out of 20 (25%) individuals receiving MSP1_42_/AS01 and three out of 10 (30%) individuals receiving rabies vaccine had activities above their day 0 baseline values. These differences were not necessarily MSP1_42_/AS01 vaccine related, and on the whole, there was not a statistical difference between mean inhibitions measured on day 70 compared to day 0 (p = 0.460). Like the US study, no detectable inhibition was demonstrated against heterologous 3D7 parasites (data not shown). When the functional antibody activities induced by both MSP1_42_/AS01 50 μg dose groups are compared, the activities are higher post-vaccination in the Kenya study, but these findings are complicated by the higher baseline levels of inhibition in that malaria-exposed population.

#### Antibody fine specificities

**Figure 6:**
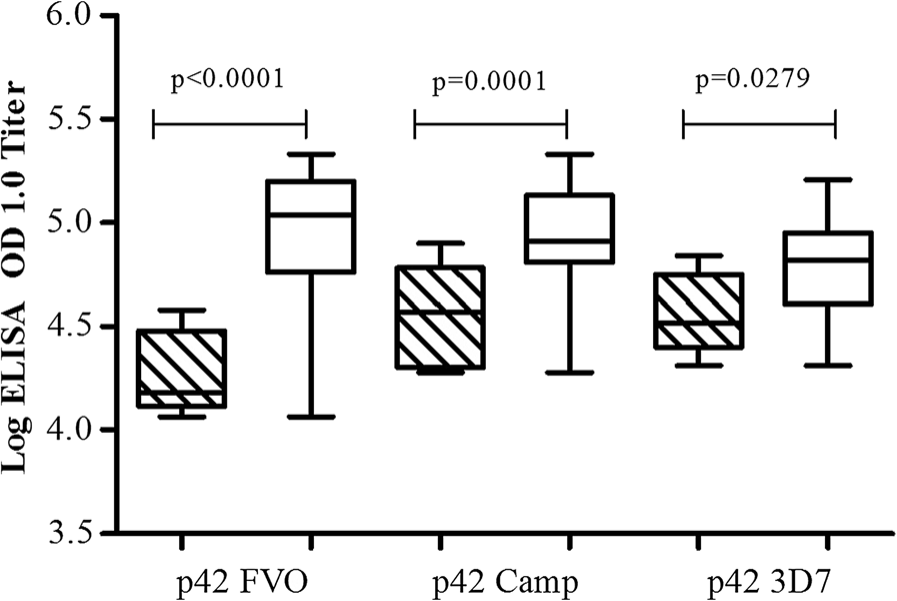
**Allele specific antibody titres to 50 μg MSP1**_**42 **_**(FVO)/AS01 *vs *50 μg MSP1**_**42 **_**(3D7)/AS02 post third immunization.** Comparison of MSP1_42_ allele specific antibody responses induced by immunization with 50 μg MSP1_42_ 3D7/AS02 and MSP1_42_ FVO/AS01. Log ELISA OD 1.0 titres are reported as the median and 25 and 75% quartile antibody response. Hatched boxes indicate the antigen specific antibody responses induced by the MSP1_42_ 3D7/AS02 vaccine, a study conducted at WRAIR in 2001 (reference 7), and open boxes indicate the responses induced by the MSP1_42_ FVO/AS01 vaccine. Plate antigens were the WRAIR *E. coli* expressed recombinant proteins, MSP1_42_ 3D7, FVO and CAMP alleles. P values are calculated from two-tailed t-tests.

For the US study, allele specific ELISAs were performed to assess the ability of the MSP1_42_/AS01 vaccine to induce cross-reactive antibody responses against the heterologous alleles of MSP1_42_. To assess allele specificity of the antibody response, sera from day 70 from the 50 μg dose group as well as from the MSP1_42_ (3D7)/AS02 study (8) were evaluated against the *E. coli* expressed recombinant MSP1_42_ alleles (MSP1_42_ FVO, 3D7, CAMP) by ELISA. The log-transformed data from these assays are shown in Figure 6 as the median and 25th and 75th quartile. No significant differences in antibody titres were detected between the 10 μg and 50 μg doses for either vaccine (data not shown); however there was a statistically significant difference in the ability of the 50 μg doses of MSP1_42_/AS01 compared to MSP1_42_ (3D7)/AS02 to induce responses to both the FVO (p < 0.0001) and CAMP (p = 0.0001), allotypes of MSP1_42_ which reportedly represent 90% of field isolates in Western Kenya. In addition, the median value of the response induced by the FVO MSP1_42_/AS01 vaccine was greater when tested against the heterologous 3D7 allotype than that induced by the homologous MSP1_42_ (3D7)/AS02 vaccine (p = 0.0279).

## Discussion

In these first-in-human studies, MSP1_42_(FVO)/AS01 was evaluated, first in 26 malaria-naïve adults in a dose-escalation fashion, followed by administration of only the 50 μg dose to 20 malaria-exposed adults with a rabies comparator vaccine given to 10 concomitantly enrolled subjects. The MSP1_42_/AS01 vaccine in both studies was shown to be well tolerated, with the solicited AEs profile similar to what has been seen with past studies of AS01 in combination with other recombinant proteins [21-24].

In subjects in the US study, this vaccine formulation proved to be equally immunogenic at both 10 and 50 μg doses, with relatively similar antibody titres between the two dosage groups at all time points. While not directly comparable due to population differences and malaria exposure, there were generally higher titres induced by the 50 μg dose vaccine in the US study compared to the same dose in the Kenya study. Given the relatively high levels of antibodies to MSP1_42_ at all time points measured in both the MSP1_42_/AS01 and rabies vaccine groups, it is possible that immune responses elicited in Kenyan adults with significant malaria exposure may interfere with the induction of antibody responses relative to the responses observed in US subjects. This issue may be less relevant for young children who have had less sustained malaria exposure and/or lower antibody titres, and thus, in this context, the vaccine may not have to overwrite clonally imprinted responses, i e, pre-existing immunity [11]. For both studies, there was a significant increase in MSP1-specific antibody titres after the second vaccination but no further boosting of responses after the third vaccination. This phenomenon has been seen with other recombinant protein antigen/adjuvant system vaccines in malaria-naïve adults [8,22], although the quality of the antibody responses with respect to avidity or subclass would require further evaluation. Antibody titres also appeared to be relatively stable through the follow-up period after the third vaccination.

The role that MSP1_19_ plays in the formation of the parasitic food vacuole [25] suggests that antibodies that are directed against MSP1_19_ may be able to interfere with the intra-erythrocytic parasite development [26]. In order to evaluate functional antibody responses induced by the vaccine, the *in vitro* GIA was employed to measure the effect of antibody on parasite growth and development. In both the US and Kenya studies, it appears that MSP1_42_ FVO/AS01 is capable of inducing some functional GIA activity in a few subjects when tested at 20% (v/v), primarily directed against the homologous FVO parasites and not the heterologous 3D7 *P. falciparum* parasites. In the previous clinical trial using the alternative allele, MSP1_42_ 3D7/AS02 [8], albeit with a different Adjuvant System, three out of five subjects from the 50 μg dose achieved similar levels of inhibitory responses against the homologous allele parasites; however, when this same vaccine was tested in 200 children aged 12 to 48 months residing in Western Kenya in a Phase 2 trial, there was no apparent efficacy [12]. Evaluation of their immune serum tested at 5% (v/v) showed no vaccine-related GIA activity; however significant inhibition was detected predominantly toward FVO allele parasites, which were not vaccine related and most likely due to natural exposure [manuscript in preparation, E Angov]. Other trials with MSP1 vaccine candidates have reported similar discordance between the magnitude of antibody titres and functional GIA activity [15,27,28].

Although the functional antibodies detected by GIA to blood-stage vaccine antigens such as MSP1_42_ have been proposed as putative surrogates of antigen quality and protective potential, there is little direct evidence supporting this concept outside of findings from epidemiologic studies evaluating the relationship of MSP1 antibody titres and functional inhibition by GIA with reduced parasite densities or clinical disease [29-33]. Studies from endemic areas suggest that the acquisition of inhibitory antibodies may be dependent upon the target antigen (MSP1, AMA1, etc.) [31], and the age of the individual whereby growth inhibitory activity had an inverse association with increasing age [29,31,32]. In a study in Kenyan children and adults, increased levels of growth inhibitory antibodies correlated with a modest delay to time to infection but only in younger children [29]. Similar findings were recently also reported for West Africa where reduced malaria risk in children and the presence of inhibitory antibodies contributed to, but were not solely implicated in, the acquisition of protective immunity [33]. Conversely, in a longitudinal study of children and adults in Kenya [31], support for a link between levels of MSP1-specific antibodies, growth inhibition and risk of symptomatic malaria was not observed. Further complicating this picture is the existence of “blocking” antibodies, which “block” the ability of “inhibitory” antibodies to act against the parasite as well as neutral antibodies, those that do not inhibit nor block [34]. Several studies report that children naturally exposed to malaria may develop both blocking and/or neutral antibodies to MSP1 in addition to inhibitory antibodies [35,36]. Thus the humoral immune response induced in malaria-naïve vaccinated subjects may differ qualitatively from those living in endemic areas who may already have established blocking and/or neutral antibody specificities.

It should be cautioned that one should not rely solely on an unvalidated *in vitro* functional assay such as the GIA when making clinical development decisions on progression of blood stage vaccine candidates. Since no surrogate markers of protection have been identified using active immunization, it is not known whether the absence of significant GIA responses in this study is meaningful. Induction of antibodies that function to inhibit erythrocyte invasion in malaria-experienced populations is influenced by many factors, including age and transmission level, as well as the heterogeneity of circulating parasite strains, and since malaria parasites appear to use redundant invasion pathways, the current GIA readout methods may not fully capture the complete effect of anti-parasite activity. The malaria vaccine researchers at WRAIR are currently re-examining the allele-specific effects from the Phase 2 study that used the 3D7 MSP1_42_/AS02 formulation in order to assess whether specific parasite genotypes in breakthrough malaria infections in the vaccinated group signified selection against the vaccine strain [12]. In addition, the evaluation of new surrogate functional assays is urgently required to assist in down selection of promising blood stage vaccine candidates. Towards this end, the WRAIR has recently improved the development of a promising new functional *in vivo* assay which measures the ability of passively transferred human immunoglobulin to protected mice challenged with transgenic *Plasmodium berghei* parasites containing the p19 of *P. falciparum* D10 strain [37]. The advantage of such a transgenic model is that immune effectors cells are present, thus enabling participation of Fc-receptor positive cells in the clearance of the parasite as proposed previously [38,39]. In addition to the current formulations, alternative vaccine platforms such as particle-based delivery and/or viral vectors could enhance the immunogenicity of MSP1 and increase its utility as a component of second-generation malaria vaccines.

## Conclusions

The results from these two first-in-human studies of MSP1_42_ FVO/AS01 are encouraging; the vaccine was of acceptable tolerability and is able to generate high titres of FVO-specific antibodies, FVO-like parasites being the predominant circulating *P. falciparum* MSP1 allotypes in western Kenya. While the GIA assay results were not as robust as had been hoped, there is still uncertainty in the interpretation of the GIA as a surrogate marker of blood stage immunity. In addition, the ability of the FVO MSP1 vaccine to induce significant cross-reactive antibodies against heterologous alleles provided encouragement for continued advancement of this vaccine candidate.

## Competing interests

MCD, OG, JC and WRB are employees of GlaxoSmithKline Biologicals s.a. (GSK). They own shares and options to shares in GSK. In addition JC and WRB are listed as inventors of patented malaria vaccines, but do not hold a patent for a malaria vaccine. Specifically EA and JC are listed as inventors on patent for this MSP1_42_FVO antigen. The Study Sponsor was the Office of the Surgeon General, US Army. This study was funded by the US Agency for International Development (USAID), with partial support from GlaxoSmithKline Biologicals (GSK) and the US Army Medical and Materiel Research Command (USAMRMC). There are no other competing interests.

## Authors’ contributions

EA and FK developed and manufactured the MSP1 vaccine antigen while MCD, OG, JC, WRB are employees of GSK who provided the adjuvant system. The studies were designed by NO, EA, OG, MK, MCD, WRB, LS, CD, JC, MP, DGH, CFO and MDS. The principal investigators were NO and MDS and MK, JB, LO, JC, BA, DT, WO, GAO, BO, MP, and CFO helped execute the clinical trials. NO, MK, LW, MCD, MP and MDS were involved in regulatory aspects of the vaccine and study. The immunological assays were conducted by EA, EBL, JB, JW, NR, BH while EA, EBL, MS and MDS were involved in data analysis All authors have read and approved the final manuscript.

The opinions or assertions contained herein are the private views of the authors, and are not to be construed as official or as reflecting the views of the Department of the Army or the Department of Defense.
